# Doctors’ Views on How to Improve Communication and Quality of Care for Patients Experiencing End-of-Life: A Qualitative Descriptive Study

**DOI:** 10.3390/healthcare9101294

**Published:** 2021-09-29

**Authors:** Fiona McCormack, Rachel Hopley, Judith Kurth, Zafar Iqbal

**Affiliations:** 1 Centre for Health and Development (CHAD), School of Health, Science and Wellbeing, Staffordshire University, Stoke-on-Trent ST4 2DF, UK; 2 Institute for Community Research and Development (ICRD), Faculty of Arts, Business and Social Sciences, University of Wolverhampton, Wolverhampton WV1 1AD, UK; r.hopley@wlv.ac.uk; 3 Public Health England, Manchester M1 3BN, UK; Judith.Kurth@phe.gov.uk; 4 Midlands Partnership NHS Foundation Trust, Trust HQ, St Georges Hospital, Stafford ST16 3SR, UK; Zafar.Iqbal@mpft.nhs.uk

**Keywords:** end-of-life care, communication, delivery of healthcare, advance care planning, qualitative research

## Abstract

(1) Background: There remains a lack of sufficient progress in enhancing quality of care for patients experiencing end-of-life. This study aimed to better understand the views of doctors on how to improve end-of-life healthcare, in light of existing challenges and processes. (2) Methods: This qualitative descriptive study used semi-structured individual interviews. Through purposive sampling, sixteen doctors from primary care (three general practices) or acute care (one National Health Service hospital trust) participated. Interviews were audio-recorded, transcribed and thematic analysis conducted. (3) Results: Two main themes were identified: First, planning for patient-centred care—conversations about end-of-life care should take place earlier to allow for care that is planned and personalised. The need for more training and improvements to documenting patient wishes were highlighted. Second, delivering on patients’ wishes: improvements to the healthcare system—the importance of a record of patient wishes that can be shared across the system was identified. Improved utilisation of available resources is also needed to better deliver quality patient-centred care. (4) Conclusion: More effective communication and coordination across acute and primary care settings is needed. The importance of patient wishes and advance care planning was emphasised. More guidance at a strategic level may help provide clarity about expectations, roles and responsibilities.

## 1. Introduction

Providing quality end-of-life care is a global public health and health systems problem [1]. Professionals and public bodies have been calling for actions to improve medical decision-making near the end-of-life over a number of years [2,3]. Despite ongoing efforts to improve end-of-life care, there are still unmet needs [4]. It has been argued that old age and end-of-life are over medicalised with an emphasis on extending life, no matter what, over quality of life [5].

Whilst definitions of ‘end-of-life’ vary, in the UK, guidance from the General Medical Council considers a patient to be ‘approaching the end-of-life’ when they are likely to die within the next 12 months [6]. The same definition was used in the first national strategy for end-of-life care in England, published in 2008 [7], and subsequent strategies, including the most recent strategy of the National Palliative and End of Life Care Partnership in 2021 [8]. The ambition is to help people to die with dignity, through good planning and good care [6]. Acknowledging that individual preferences may vary, the strategy sets out the following as constituting a ‘good death’: being treated as an individual, with dignity and respect; being without pain and other symptoms; being in familiar surroundings; and being in the company of close family and/or friends [7] (p. 9). However, since the 2008 strategy was published, there have been at least 48 further reports on palliative and end-of-life care in the UK, with much consensus on the main issues and priorities but a lack of progress made [9,10].

Advance care planning (ACP) was recommended by the first UK strategy, which prompted much attention [11]. ACP is the process of discussing and recording a patient’s wishes around goals of care for patients who may lose capacity or the ability to communicate in the future [12]. A systematic review reports that ACP is often found to decrease life sustaining treatment, increase use of hospice and palliative care and prevent hospitalisations; it should be noted that most papers in that review were from US (81%), with different legal systems, healthcare and cultural contexts to the UK [12]. Similar challenges and debates also seem to prevail about ACP in the wider literature. For example, more recently, it was highlighted that further research with specific population groups would help inform how to improve patient engagement with ACP, and emphasised that discussions should be part of health promotion efforts with a focus on planning for a comfortable and peaceful death [13].

In the UK, the Gold Standard Framework (GSF) was developed, initially in primary care and then care homes, to support teams working in the community to deliver proactive, coordinated care for their patients [14]. The aims of GSF set out recently are to improve: the quality of care experienced by people in their last years of life, co-ordination, communication and teamwork, outcomes for people (e.g., to die where they wish and reduce hospitalisation), and outcomes for healthcare systems from more cost-effective and appropriate use [15].

Despite the introduction of ACP and GSF, challenges remain for both primary and acute care to deliver quality end-of-life care. GPs (general practitioners) in England were found to be generally supportive of the need to have ACP discussions with their patients. Yet, difficulties with the capacity of the existing health and social care system (such as limitations of the community services available) may discourage them from having these discussions fully, as there was concern about raising unrealistic expectations for patients that may not be deliverable due to a lack of services in other parts of the system [16]. As such research that focusses generally across the system is warranted.

Certainly, providing end-of-life care within the acute hospital setting appears to be challenging. There is evidence from the last ten years that quality of end-of-life care within the hospital setting remains sub-optimal [17,18]. Research with patients and families about care in hospitals suggests patients receive poor management of symptoms and that family members can find it difficult to engage with health professionals in making decisions about care and management of end-of-life [19]. Various studies have identified communication issues as a fundamental challenge, both within the hospital setting and more generally [18,19,20,21,22,23]. Clear, comprehensive and timely communication is crucial to facilitate a ‘good death’ [22], and prevent inappropriate treatment at the end of a patient’s life [21].

The nature of acute hospital environments can present challenges for caring for dying patients. With the multi-bedded rooms and high patient turnover of hospitals, it may be unhelpful to make direct comparisons with other settings in terms of standards of care [19]. Other key barriers include the difficulty in ‘standing back’ (i.e., the opportunity to pause and reflect) within the acute hospital, and that professional hierarchies restrict the input that junior/nursing staff can have into decisions [20]. That services remain ‘fragmented’ presents challenges [24]. Issues around continuity of care in hospitals and after discharge have been highlighted [18]. Patients at the end-of-life may be admitted to hospital due to difficulties in finding an alternative solution, for example, a lack of planning which may have enabled them to remain at home if they wished [21].

Previous studies have tended to investigate end-of-life care in specific conditions/patient groups. In contrast, this research focused more generally on the views and experiences of doctors working within acute and primary care in a specific locality within the West Midlands of England. This setting was chosen because of specific interest in this topic from the NHS (National Health Service) Clinical Commissioning Group (CCG) and the local authority’s Medical Director and Director of Public Health. This paper reports suggestions for improvements to enhance both quality of care and patient experiences of end-of-life care. It emphasises the need for more effective communication and co-ordination across acute and primary care systems, and advance care planning so that end-of-life care conversations happen earlier with patients, discussing and reviewing their preferences and wishes. These preferences need to be documented and easily accessible to the patient, and everyone involved in their care, across the different systems.

## 2. Materials and Methods

### 2.1. Study Design

This descriptive qualitative study [25] investigated the general views and experiences of doctors in relation to the delivery of end-of-life care in a specific area of the West Midlands of England. Semi-structured interviews were conducted with 16 doctors who worked within acute and primary care in the area. Data collection took place between August 2017 and February 2018.

### 2.2. Participants

Doctors were recruited from primary and acute care using a purposive recruitment strategy to invite the full spectrum of doctors to take part, including those working in varying specialisms and with varying years of experience. The Medical Director and local CCG emailed all doctors in acute and primary care settings, respectively, with a participant information sheet to inform them about the research and invite them to contact the researchers directly to express an interest in joining the study. Whilst 19 doctors expressed an initial interest, three were uncontactable thereafter. This provided a convenience sample of 16 doctors.

The following information is provided to describe the characteristics of the sample. The participants (6 male and 10 female) were aged between 27 and 63 years and had been working in their role for between 1 month and 30 years (see Table 1 for further details). As Table 1 shows, the sample captured a range of specialisms and levels of seniority.

### 2.3. Data Collection

Individual one-off interviews were conducted by the second author (R.H.) and a research assistant using a semi-structured interview guide. The interview guide was developed and refined with a steering group, which included consultants and GPs involved in end-of-life care. Participants were invited to describe their role, and subsequent questions explored their views and experiences of providing end-of-life care, specifically how they uncovered information about patient wishes, what challenges they faced as a provider of end of life care and suggestions for improvements. Interviews took place in private at the participant’s workplace or the researcher’s workplace (University) according to participant preference. The interviewers were experienced in health-related research and qualitative interviewing, and had no prior relationships with any participants. The researcher explained the purpose of the interview prior to obtaining written informed consent and commencing the interview. Interviews lasted between 15 and 60 min (mean = 37 min). Interviews were audio-recorded and transcribed verbatim.

### 2.4. Data Analysis

An inductive thematic analysis [26] was conducted and the qualitative software package QSR NVivo 11 (QSR International Pty Ltd., Doncaster, Australia) was used to help manage the process. All transcripts were coded before the researchers identified, refined and finalised the themes. To minimise potential bias in interpretation, the first and second authors (F.M. and R.H.) coded the data separately, identified themes and wrote preliminary findings. Two co-investigators (Z.I. and J.K.) helped refine themes. Findings were shared with the steering group for the purpose of obtaining their feedback, and at a local End-of-Life Programme Meeting comprising representatives from the CCG, acute and primary care, and local hospices. The feedback received and subsequent conversations provided useful context for the themes identified and have informed the discussion and implications of the findings in this paper.

### 2.5. Ethics

Ethical approval for this study was obtained from the University Health Sciences Ethics Committee (approval granted on 14 June 2017) and NHS Trust (Health Research Authority—IRAS Project ID 230859, approval granted 4 August 2017).

## 3. Results

Two main themes were identified, as shown in Table 2.

### 3.1. Planning for Patient-Centred Care

Participants explained how conversations about end-of-life care should take place earlier to ensure care is planned and personalised as far as possible.

#### 3.1.1. Preparing for End-of-Life Care Conversations

Participants frequently referred to end-of-life care conversations happening “almost too late” (Acute Care 01), and the need to recognise end-of-life and initiate conversations with colleagues, patient and families earlier:

*“Patients come to me having had a lot of input from colleagues over a long period of time and no-one has had the brave discussion. The one that needs to be had, which is that sometimes it is kinder and more loving to withdraw care than it is to press on.”*

(Acute Care 02)

*“It is really important to capture it when they are of sound mind. For me, that is a challenge, having a conversation with someone who is clearly going into cognitive decline and I always sort of come away feeling I wish I could have had the conversation earlier.”*

(Primary Care 01)

The implications of delayed conversations were described as “depriving people of the right to choose how they want to die” (Acute Care 08), and families losing the opportunity for quality time with the patient. Participants were highly critical of this avoidance and its implications for the patient and their care.

Participants identified a range of challenges to having timely conversations. They emphasised that it can be difficult to predict outcomes as “every person is different” (Acute Care 10). This could make it difficult to reach an agreement within the medical team which often raised fears of failure (i.e., getting a prediction wrong, of interfering and/or contradicting other staff, and of potential repercussions–legally and to their reputations).

Within the acute setting, it was suggested that readiness to identify a patient as end-of-life could vary by role/level of contact with the patient. One participant reported that they often identify a patient as requiring end-of-life-care earlier than their colleagues because of first-hand experience of seeing the “suffering that you inflict on the patient” otherwise. “How close you are to the patient” was also considered to be important:

*“The nursing staff often feel we should stop when the intensive care doctors don’t feel that yet because we obviously are not that close to the patient as it is the bedside nurse that is physically at the bed side all the time. Therefore, we often say we can still try a bit longer; we can still try this or that; and the doctors that only come occasionally are even more remote or further away from the actual suffering in front of them, they tend to push even longer sometimes than we do.”*

(Acute Care 01)

Participants identified challenges around finding appropriate time and space to have such conversations:

*“… it requires time and if anything, I don’t think we get enough time to do that because there is so much more expected of us. In that way, if anything can be improved—yes, give them quality and time”*

(Primary Care 02)

The importance of timing for the professional was also highlighted:

*“Sometimes from my side as well obviously there are days where I feel more capable of talking about things. There’s other days where I feel, you know, sort of less inclined to tackle something sort of head on, where I would like to avoid conflict a bit, because it’s not a great day for me either.”*

(Acute Care 10)

Generally, participants reported feeling confident and comfortable discussing end-of-life care having built up their own routine/technique over time and with years of experience. They identified a need for medical professionals, and younger professionals in particular, to be more confident at having such conversations. The need for education and training related to providing end-of-life care, and communication skills, was emphasised:

*“… we just have to keep the momentum and continue to make sure that we train everyone, and everyone* *has got access to communication skills training, that people are supportive when they are sort of having to make difficult conversations … that the focus on what’s important to the patient stays.”*

(Acute Care 10)

One of the junior doctors highlighted that they do receive support from senior staff around having end-of-life conversations and that if they are finding it too difficult, they can still “observe and learn” rather than lead the discussion. The other described personally seeking out a Palliative Care Nurse to learn more about end-of-life care because it was a topic of specific interest to them. Noting that relatively few of their counterparts would have a placement with a hospice, one of the junior doctors thought it would be useful for more training to improve understanding about hospices and what they are able to provide in relation to end-of-life care:

*“…I was just mentioning to my seniors that maybe we should have a team who can have, maybe a half hour meeting, or we have got these junior doctor forums and discussions every week, where maybe a representative can come down and tell us what exactly happens in the hospice so that everybody will know what the hospice does; not just in the community but also in our own circle”*

(Acute care 07)

#### 3.1.2. Planning for a Personalized Journey

Here, improvements related to planning and documenting patient’s wishes for their end-of-life care. With an emphasis on the patient, participants suggested a shift was needed towards more holistic end-of-life care and ensuring that care was person-centred, focusing on the patient as a person, rather than the condition or medication. This included acknowledgment, understanding of, and sensitivity towards their beliefs, hopes and wishes for care. A Paediatric Consultant recommended that living and dying should be planned for simultaneously:

*“… we use this thing called parallel planning. We plan about end of life, but we also plan about living—living and symptoms, because actually they both happen at the same time.”*

(Acute Care 05)

In addition, it was suggested that the language used in discussing end-of-life care with patients and families is revisited to be more appropriate:

*“I don’t know if we should say ‘planning for our future care?’ Is it advanced care plan… but not making it so scary?”*

(Primary Care 02)

Overall, there were calls for more advanced care planning as a concept, and more formal documentation of some description. Some participants acknowledged the fear of inflexibility with formal advance care plans (ACPs) and that patient’s wishes might change. However, the majority highlighted that such a plan is helpful as a guide to inform decisions.

From the participants involved in neonatal/paediatric care, there was a strong sense that ACPs are used effectively to facilitate timely—and importantly, ongoing—conversations with parents about their wishes for their child’s care; they felt this often marked them apart from how adult end-of-life care may be approached. For instance, some participants were not aware of ACPs and/or referred to the local hospice/palliative care teams at this point.

Participants felt that discussions about end-of-life care are more effective (i.e., patients open up about what is important to them, how they feel and what they hope for) when there is a relationship and trust. This was considered important particularly because of the subjective nature of ‘quality of life’:

*“We can’t really put ourselves in the shoes of a patient whose life is having visitors, watching TV and reading a book. That’s a good quality of life for that person and we are thinking, well we can’t walk to the shops so…I don’t think we know and I think it has got to come from the patients”*

(Acute Care 06)

Often for participants, this raised questions about what quality healthcare looks like for patients approaching end-of-life, and how ‘success’ is measured in such circumstances. Many called for a shift in focus from prolonging life to ensuring the patient’s death (and life) is dignified:

*“Sometimes we can’t cure everyone … but where I can succeed is ensuring that the child’s death is dignified: that their symptoms are well controlled and that they are able to achieve what they are able to achieve, however small those wishes might be—enabling that to happen”*

(Acute Care 05)

### 3.2. Delivering on Patients Wishes: System Improvements

Another set of improvements related to how to overcome existing system challenges, to maximise its ability to deliver quality end-of-life care that is responsive to patient wishes. It was emphasised that there is a need to go beyond having conversations, to actually put mechanisms in place that will enable wider improvements to the quality of care available to patients at the end-of-life.

#### 3.2.1. Sharing and Communicating Information

The importance of having a record of the patient’s wishes that is readily available and easy to access was emphasised. Participants highlighted that, at present, this is not the case within the system itself and that the onus would be more on the patient, their family or carers to make those wishes known:

*“As an emergency physician, I can tell you most of the times the information is not easy to access if it is there, and unless the patient brings it himself, or the family or the carers are quite aware of it, you don’t know where to find it, you don’t know how to access it.”*

(Acute Care 08)

There was wide reference to things being ‘slow’, and that crucially, in the absence of such information, the focus would be to “carry on and carry on” and treat the patient (Acute Care 08). Participants felt the document should be accessible to anyone involved in providing care/treatment to the patient. However, a key challenge raised here was that primary and secondary care have different IT systems and that information is not shared between them:

*“We need really a single agreed care plan for a patient, which not only is shared…held by the patient…but is easily shared with the all the partners and stakeholders and really the way to do it is to have an electronic record.”*

(Primary Care 01)

There were different views on whether paper or electronic copies would be most useful and often a combination of the two was suggested with the patient having a copy with them at home (to help paramedics/ambulance know of wishes), as well as a copy with their GP/on file accessible to all those involved in providing end-of-life care to that individual. There was a suggestion of implementing something similar to the traditional pregnancy folder notes that are intended for the patient to carry with them at all times. There were also suggestions that explicit documents containing patient wishes would improve transitions between hospices, care homes and hospitals.

#### 3.2.2. Process and Resources

Other improvements related to the provision of resources (including staffing and processes) that would enable patients to avoid unnecessary and/or unwanted admissions to hospital during end-of-life. For example, district nursing support, palliative care nurse input, and hospice spaces were all identified as important. There was acknowledgement that resources are tight and often the focus in hospitals is on beds for those patients who they can help to survive or those who are going forward with organ donation. One participant suggested having a special palliative care ward within the hospital, highlighting that whilst there are palliative consultants at present, there are not palliative care beds.

Improving hospital discharge in terms of planning and speed, rather than avoiding hospital admissions was also recommended. This participant believed admissions are often appropriate for palliative care patients—for example, if they need to have a scan/test/infection treated:

*“But I think that where the hospital is then lacking is about getting them out quickly. … From my point of view, rather than focusing too much on admission avoidance, I would focus more on sort of a quicker discharge.”*

(Acute Care 10)

Related to this, another improvement put forward was to expand who is able to prescribe medication at home:

*“Bureaucracy of the system means only a GP can prescribe in the home”.*

(Acute Care 13)

A recurring suggestion was to have a co-ordinator with overall responsibility to make sure ‘everything runs smoothly’. This designated person would co-ordinate all services involved to meet the needs of the patient and give clarity to roles and responsibilities. Therefore, it would not just be end-of-life discussions that would be patient-centred, but care too. Having a designated co-ordinator would also help continuity of care which was described as important for the professional and patient in ensuring a compassionate and informed approach to an individuals’ end-of-life care:

*“So, for me it is sides of a triangle which consists of integration, continuity of care and co-ordination of care. If we can get those three things right, I think we will get somewhere.”*

(Primary Care 01)

## 4. Discussion

For doctors working in acute and primary care in the West Midlands, it is clear that end-of-life care is challenging and complex. The need for health professionals to ‘be brave’—to recognise end-of-life and initiate such ‘difficult’ conversations earlier was evident. Although clinicians face many barriers to such conversations (such as uncertainty around prognosis, fear of causing distress, navigating patient readiness and feeling unprepared), patients and relatives value sensitivity and empathy which are core, non-specialist elements of communication skills across all medical specialisms [27].

Participants acknowledged the challenges, but also the importance of a proactive approach so patients are given the right and ability to make decisions about their wishes whilst they have capacity. A simple opening question of “What’s important to you?” can open the discussion and help to ensure people have choices and input into their care that are focused around their own individual needs [28]. Initiating such a conversation early in the patient’s end-of-life journey would help empower patients and improve the quality of the care they experience [27]. Our findings support the notion that such an approach to communication could reduce hospital admissions in the last three months of life [29]. It is also important to do this in a timely manner as this can impact the support and resources available to the patient [30].

The need for more education around recognising patient deterioration, the dying process and end-of-life care was identified and supports existing literature [22]. ‘Training’ should address fears and the confidence to have conversations earlier, as well as developing skills, which could be most effective through workplace learning [24]; such as shadowing, peer-to-peer training, workshops delivered by the palliative care team and the more formal advanced communication training referred to by participants. For more formal training interventions focused on communication in end-of-life care, there is a need for improved clarity and consistency in how they are reported and assessed for effectiveness [31].

There are opportunities to improve the sharing of knowledge and processes and learn from others across the system (including hospices and charities). For example, hospices locally deliver training for free around having difficult conversations and building confidence. Meanwhile, the insight and skills developed by staff working in paediatrics around developing relationships and working with patients/families to provide dignified and compassionate patient-centred end-of-life care, and the valuable resource that Palliative Care Consultants can provide related to end-of-life care queries could be better utilised. This could ensure patients’ wishes are considered broadly across all aspects of healthcare and not limited to palliative care.

The potential for nurses to have a stronger role warrants attention [32]. Participants suggested that nurses spend more time with the patient and may therefore recognise deterioration in patients sooner than medical consultants. This is important because nurses tend to have limited input into such discussions and decisions [20]. How to build on the strengths within the system and work together across different disciplines, roles, and levels of seniority is needed to improve delivery, provide more holistic end-of-life care, and reduce the over-medicalisation of end-of-life that many participants referenced.

Participants emphasised the importance of patient wishes. With the subjective nature of quality of life and the ‘inexact science’ of predicting outcomes, participants often felt that decisions should come from the patient and/or their families. The potential for the concept of advance care planning was something participants felt would help facilitate this, but that this must come with clear opportunities to revisit and update the associated documentation because patient wishes, as well as health needs, may change over time [16,21]. There were calls to amend the language used to make it easier for patients to understand—e.g., planning for future care, rather than advance care planning. It should be made clear to patients, families and healthcare staff, what aspects are legally binding. Participants highlighted that ultimately, decisions would depend on medical opinion and available resources alongside patient wishes.

In addition to implications for the healthcare system, there are also important considerations for public health. This is crucial in the current context of excess mortality due to COVID-19 in England and the strain on palliative care services [33]; with excess mortality due to COVID-19 following the social gradient in England [33], there is a potential impact of widening health inequalities and potential regional variation in increased demand for end-of-life care. Even prior to the pandemic, future projections for palliative care identified increasing need, and urged that healthcare systems need to implement changes now in anticipation of the future age-related growth in deaths from chronic illness [34]. In that study, dementia and cancer were identified as the main drivers of increased need [34]. There has also been recognition that a greater number of conditions require palliative care approaches, including end stage heart failure and chronic obstructive pulmonary disease (COPD). Against this backdrop, there is a clear need to normalise end-of-life discussions in the general public (as well as between doctors and patients), which would help people and their families/those important to them to think about what their wishes might be for future care.

How to share patient wishes/documentation across the healthcare system requires attention. Some participants (excluding palliative care and paediatrics/neonatal staff) highlighted that they would not necessarily know where to look to see if an ACP was in place and that the paperwork can be slow. That information cannot be shared across primary and secondary IT systems needs to be addressed. Participants talked about a ‘fragmented system’ and the need for a single agreed care plan that is held by the patient and can be shared with all those involved in delivering care. Previous UK research that explored the successes and failures of an Electronic Palliative Care Coordination System highlighted that research is lacking about integrating data sharing initiatives and also the challenges of developing and implementing such systems [10].

Participants stressed the need to go beyond having conversations and obtaining patients’ wishes. They identified a range of issues that could prevent end-of-life care being delivered in accordance with a patient’s wishes and called for processes to be put in place to improve the system. Commissioning guidance published in 2016 could be useful for this purpose [35]. This echoes findings of a larger qualitative study where the majority of participants (95 health professionals working in dementia and palliative care in the North East of England) were uncertain as to whether existing service provision could meet patients’ wishes [36]. Our study suggests a need for more strategic guidance within local CCGs to address such concerns, and to provide clarity about expectations and legal implications as well as roles and responsibilities. Such guidance is required to help support staff to ‘be brave’. Arguably, it is important to gain patient and families perspectives to help inform this.

Some limitations to this study must be acknowledged. It is possible that doctors interviewed were more enlightened about end-of-life care, and thus volunteered to participate, although from the range of opinions gained we are confident we have not been too limited by this. This study captured the views of a self-selecting, convenience sample of doctors across acute and primary care from one area in the West Midlands, UK. Given the exploratory nature of the study, a non-probability sampling strategy was used. As such, the findings cannot be considered representative of that wider group of doctors, nor on a wider scale. Further qualitative studies are required to explore how the findings apply to others involved in delivering end-of-life care (e.g., other specialists, other health professionals including nurses, and other sites and settings including hospices). More research is also needed around who would be the best place to fulfil the role of coordinator that interviewees felt was required. An NHS guide to coordinating care published in 2018 may provide a useful starting point for such discussions to take place [37].

## 5. Conclusions

This research adds to a growing body of evidence highlighting the challenges needed to overcome to improve communication and quality of care for patients experiencing end-of-life. It extends this by setting out suggestions for improvements, as identified by the doctors interviewed. This study demonstrates the need for better communication and coordination between professionals in various settings, to better understand, promote and utilise available assets and resources (including knowledge, advice, training and other opportunities for learning, e.g., from palliative care teams and hospices, those in paediatrics, and varying levels of seniority) across the sector as well as about individual patient-centred care. It outlines implications for the system as a whole—a need to work more effectively across the system, for more guidance at a strategic level and processes to be put in place to support this and to help staff in both acute and primary care to ‘be brave’ in discussing and providing end-of-life care.

## Figures and Tables

**Table 1:** Participant characteristics (*n* = 16).

Job Title	Specialism	*n*
Consultant (acute care)	Paediatrics	3
	Palliative Care	3
	Cardiology	2
	Critical Care	1
	Geriatrics	1
	Neurology	1
Junior Doctor (acute care)	Accident and Emergency (A&E)	1
	Generic	1
General Practitioner (GP) (primary care)		3

**Table 2:** Findings.

Theme	Subtheme
(1) Planning for patient-centred care	(a) Preparing for end-of-life care conversations
	(b) Planning for a personalized journey
(2) Delivering on patients wishes: system improvements	(a) Sharing and communicating information(b) Processes and resources

## Data Availability

Data not available due to ethical/consent restrictions in this qualitative study. Due to the nature of this research, consent was not obtained from participants for their data to be shared or made publicly available.
